# The Feasibility of Contrast-to-Noise Ratio on Measurements to Evaluate CT Image Quality in Terms of Low-Contrast Detailed Detectability

**DOI:** 10.3390/medsci8030026

**Published:** 2020-07-06

**Authors:** Haney A Alsleem, Hussain M Almohiy

**Affiliations:** 1 Departments of Radiological Science, Colleges of Applied Medical Sciences, Imam Abdulrahman Bin Faisal University, Dammam 34212, Saudi Arabia; hsleem@iau.edu.sa; 2 Departments of Radiological Science, Colleges of Applied Medical Sciences, King Khalid University, Abha 62529, Saudi Arabia

**Keywords:** radiation dose, contrast-to-noise ratio (CNR), low-contrast detail (LCD), multidetector computed tomography (MDCT)

## Abstract

**Background:** To evaluate contrast-to-noise ratio (CNR) measurements in assessing image quality, in the context of the detectability performance of low-contrast detail (LCD), in computed tomography (CT) images, since exposure to elevated ionising-type radiation is considered to present excessive carcinogenic risk, whilst also causing distress in study subjects. **Methods:** An LCD phantom module (CTP515) was utilised in the study. Three dissimilar contrast items were used to analyse the ramifications of the proportions of an object on the CNR. Three multidetector CT (MDCT) scanners were used, with 16-MDCT, 64-MDCT and 80-MDCT frameworks, respectively. The CT scans were recreated using three dissimilar remaking algorithms—soft, standard and lung. The effects exerted on the CNR by various remodelling algorithms, as well as the contrast of various objects along with the size of the objects, were explored. The Hounsfield units of each chosen object (one unit representing the outer portion of the object) and the background and the standard deviation of the noise parameter were quantified, and algorithms were developed using MATLAB. **Results:** The CNR information was greatly influenced by changing the image recreation calculations and was very much increased in the soft-tissue recreation images using 16-MDCT and 64-MDCT. The CNR information was also increased more in the optimum recreation images than in the reproduced images from the computational procedure used in the 80-MDCT. The results did not show any remarkable contrasts in the CNR values between the different object sizes. Overall, a higher kVp produced an improved CNR in all the CT scanners. In particular, there were prominent upgrades in the CNR information when the kVp was increased from 80 to 120. Higher mAs levels gave better CNR values overall, especially for greater section thicknesses. Based on the CNR estimations, the 64-MDCT provided the best correlation among the CT scanners. **Conclusions:** The objective LCD appraisal method, based on CNR measurements, was confirmed as being useful for checking the different impacts of kVp, mAs and section thickness on the nature of the picture. This procedure was similarly viable in assessing the impacts of the different reconstruction calculations and the different differentiation questions on the nature of the image.

## 1. Introduction

A significant increase in the number of computed tomography (CT) scanners has highlighted the need to reduce the amount of radiation used in them [1]. While the evolution of the CT technique has resulted in the enhanced diagnosis of disease, its disadvantage has been the corresponding radiation dosage [2]. Numerous analysts have suggested rational scan protocols for regular CT investigations and expressed a desire for the various manufacturers to guarantee high image quality using less radiation [3,4]. Consequently, radiation doses can now be lowered without sacrificing diagnostic details in the image [3]. One solution for improving image quality at low-radiation levels has relied on progress being made in the rebuilding methods [5,6,7,8], among which iterative reconstruction (IR) algorithms are commonly used. In addition, several studies have recommended that low-contrast detail (LCD) detection capability might be the most suitable method for standardising images [9,10,11]. LCD refers to the capability of a CT scan to differentiate between various objects that have similar X-ray attenuation coefficients. The most serious and noteworthy challenge in this aspect is that techniques utilising low dose decline the detectible performance of LCD, uniquely in abdomen examinations. For example, carcinogenic disease of the liver is customarily demonstrated as low-attenuation lesions inside a background of slightly higher attenuation normal tissue. Detectible capacity of LCD must be preserved with any dose-reduction strategy. Contrast-to-noise ratio (CNR) is an important tool used to determine image quality. It is a measure of image quality established on a contrast. Prior studies have shown that images obtained using hybrid-type IR (HIR) algorithms, which comprise the majority of various accessible IR procedures, are able to maintain image standards using a decrease in radiation of 20–25% to 65–67%, although some image noise and artefacts remain [12,13]. Recently, iterative-type model reproduction (IMR), an information-based IR calculation, has enabled an additional decrease in radiation dosage with enhanced image representation, although this has resulted in a notable decrease in contrast and a suppression of artefacts that are differentiated into filtered back projection in the period.

Filtered back projection (FBP) and HIR calculations in LCD scans [14,15,16,17,18]. The two foremost methods—subjective and objective—are accessible for computing the LCD of various CT images [19]. The subjective technique depends on human perception, and the objective one is based on quantitative measurements of contrast-to-noise ratio (CNR) [9]. Because of the subjectivity of human observers, the objective way is considered to be more suitable for dealing with estimating LCD execution. In this study, we aimed to assess the impacts of the settings—essentially, milliampere-second (mAs) and Kilovoltage peak (kVp)—on the LCD of CT scanners. The strategy used was based on the CNR estimations of scanned objects. We also examined the differences between different CT scanners, in terms of the CNR estimations of those scanned objects. Our overarching aim was thus to evaluate the methodology used in CNR measurements in order to assess image quality in terms of the detectability of LCD in CT images.

## 2. Materials and Methodology

### 2.1. The Phantom Model

**Figure 1:**
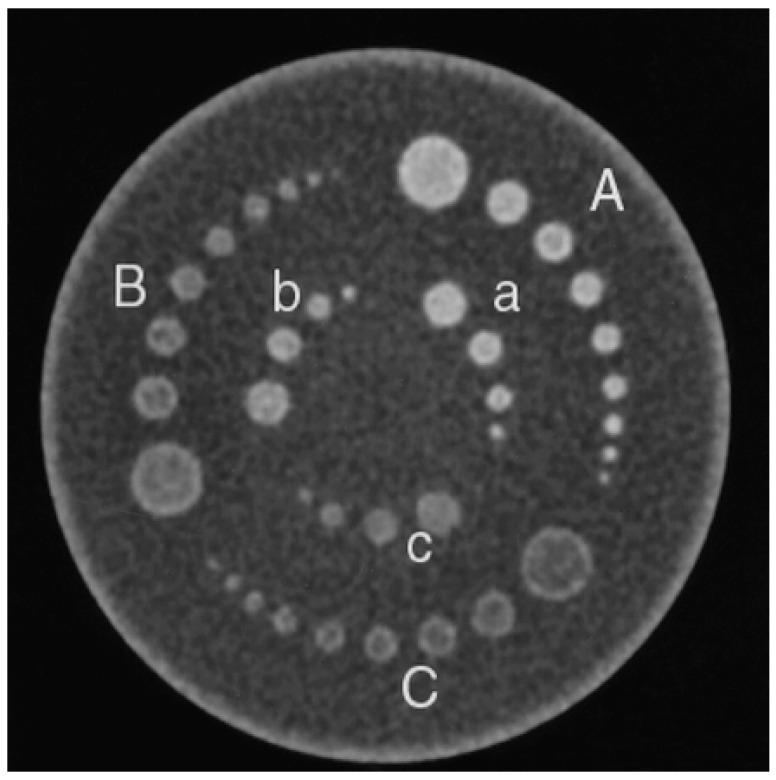
A Phantom of low contrast module. Six different regions have been depicted where low contrast objects are put (A–C and a–c). Low cylindrical objects were placed in Regions A–C to contrast the difference in the objects in Regions A/a, B/b and C/c with the backgrounds of 1%, 0.5% and 0.3%, respectively.

The Catphan^®^ 600 (Phantom Laboratory, Greenwich, NY, USA)—known as the phantom—was mainly used in this study (Figure 1). In this, the image is developed from a medium of solid cast, produced using modules with a diameter of 15 mm. Each module assesses particular audits that correlate with the usage capability of the multidetector CT (MDCT). An LCD image module (CTP-515) was installed in the phantom for use in the experiment (as shown in Figure 1). The image shows a collection of sets of round, empty, low, multifaceted natural objects, arranged on two levels in the phantom. The objects were 40-mm high, were separated by different spaces (i.e., 2–9 and 15 mm) and were examined at three contrast levels (0.3%, 0.5% and 1%). The objects arranged around the outer level were chosen for study, those measuring 5–15 mm at a 1% contrast level, those 6–15 mm at a 0.5% level and those 7–15 mm at 0.3%. Thus, three specific contrast levels were used to examine the objects to assess the effect of object size on the CNR. The objects chosen for this part of the study were 1% separate level things for two purposes.

### 2.2. The CT Scanners

Three MDCT scanners—a 16-MDCT structure (GE Lightspeed VCT; GE Healthcare, Milwaukee, WI, USA) a 64-MDCT structure (Light Speed VCT, GE Healthcare) and an 80-MDCT structure (Aquilion-80, Toshiba, American Medical Systems Inc., Minnetonka, MN, USA)—were used (as shown in Table 1). All systems were well maintained. This ensured that the scanners’ capabilities met the manufacturers’ specifications.

### 2.3. Image Acquisition

The unit of the LCD belonging to the particular phantom was assembled inside the scanner portion of the gantry. The complete measurements were made utilising two voltage increments of 80 and 120 kVp at various levels of mAs and different section thicknesses as well as reconstruction computations (as depicted in Table 2). The field of view was set at 360 mm for the 16-MDCT and 64-MDCT scanners and at 240 mm for the 80-MDCT scanner for data collection. The data were then matched up, using three specific changing configurations—standard being the initial one, soft tissue being the second and lung being the last. The effects on the CNR of the various multiple counts and separate objects, together with object size, were examined, as was the impact of kVp, mAs and section thickness.

### 2.4. Calculation of the CNR and MATLAB Software

The universal Hounsfield units (HU) of each chosen object (i.e., the outer-level articles), the background and the standard deviation (SD) component (i.e., noise) were evaluated. The required calculations to process the CNR data were made using MATLAB (v.7.14, MathWorks, Natick, MA, USA). The calculations were then applied to each image, and the CNR for everything was resolved using the equation [20] below. The SD of the mean CNR was also computed.

(1) $$\mathrm{CNR}\;=\frac{\mathrm{CT}\;\mathrm{value}\;\left(\mathrm{object}\right)-\;\mathrm{CT}\;\mathrm{value}\;\left(\mathrm{background}\right)}{\mathrm{SD}\;\left(\mathrm{noise}\right)}$$

### 2.5. Statistics and Analysis 

The Gaussian statistical distribution formula was used to determine the dispersion ordinariness of the arithmetic for each factor. Gaussian dispersion examines the likelihood of whether the arithmetic for each factor remains between two genuine cut-off points [21]. A two-route between-bunches examination of difference (using SPSS programming) enabled an interrogation of the data. An analysis of the variance (ANOVA) of the two-way-measurement analysis was performed to look at the effects of the various CT convention parameters, including reproduction of the calculations, the mAs, kVp and section thickness, on the CNR measurements of each image and each object in the images. The effects of different factors, such as object size, object level and type of scanner, were also examined by two-way ANOVA. The two-way ANOVA was utilised to calculate whether notable contrasts subsisted between kVp bunches revealed between similar types of mAs and section thicknesses, between interconnecting mAs bunches at equivalent kVp and section thicknesses and between section thickness bunches at analogous mAs and kVp. A similar type of analysis was also used to investigate whether there was a critical correlative impact between these factors. A *t*-test, at an alpha estimation of approximately 0.05, was used as part of the two-way ANOVA computations to determine whether centrality contrasts existed between the various groupings [21].

## 3. Results

**Figure 2:**
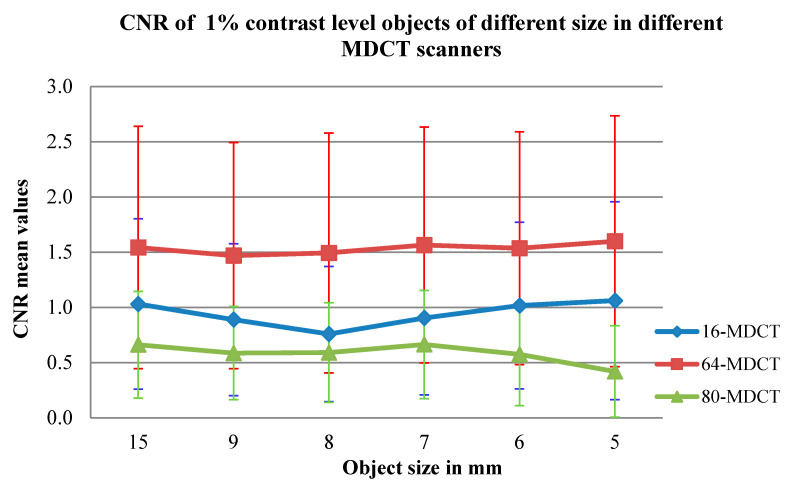
Limited effects of the size of 1% contrast levels objects on CNR values for all CT scanners. (Note the change in CNR values for 16-MDCT with 8-mm object size and for 80-MDCT with 5-mm object size.)

The effects of the size of the objects, with 1% contrast level, on CNR values were evaluated for different MDCT scanners, reconstruction algorithms, object contrast levels and mAs selections (Figure 2 and Table 3 and Table 4). Changes in the CNR values for 16-MDCT with 8-mm object size and for 80-MDCT with 5-mm object size can be seen in Figure 2. Data from the soft-tissue and reproducing-algorithm images and objects at 1% contrast are recorded in Table 3 and Table 4.

The effects of object size on the CNR values were examined at first. Object size in relation to different scanners, reconstruction calculations, levels of object contrast and mAs was also scrutinised. The effect of the reconstruction calculations, mAs, kVp and section thickness on the CNR data were then assessed. The output of the various scanners was then compared, based on the CNR values.

### 3.1. Effect of Object Size on CNR Values

The effect of object size on the CNR values, at a 1% contrast level, was assessed for each MDCT scanner, for the various reconstruction calculations, contrast levels on the objects and mAs values. It was found that negligible variations in the mean CNR values occurred for the objects of various sizes across all scanners (*p* value > 0.1) (see Table 5), but there were considerable variations (*p* value = 0.021) between the 5- and 8-mm objects at 1% contrast in the 16-MDCT. In the 64-MDCT, there was always a negligible difference in CNR values among the objects of different dimensions. In the 80-MDCT, there were significant contrasts in the CNR values between a 5-mm object and the 8- and 15-mm objects.

### 3.2. Impact of Image Reconstruction Calculations on CNR Values

In the 64-MDCT and corresponding 16-MDCT, the CNR values for the reconstructed soft-tissue images were substantially higher than those for the corresponding lung images (*p* value < 0.001). The soft-tissue images had impressively higher CNR values than the standard images in the 16-MDCT and 64-MDCT (*p* values = 0.011 and <0.001, respectively). In the 80-MDCT, however, the standard images had much higher CNR estimates than the other types of images (*p* value < 0.001). 

### 3.3. Impact of kVp on CNR Values

Using a higher kVp gave higher CNR values in all CT scanners. There were critical increases in the CNR values when the kVp was increased from 80 to 120 (Table 6). On the other hand, there was little separation between the CNR values at levels of 80 and 120 kVp at 10 mAs and corresponding 0.625-mm section thickness (*p* value = 1) within the 16-MDCT. Considering 100 mAs and corresponding 0.625 mm, there were, however, inconsequential distinctions in the CNR values when the kVp was increased to 120 (*p* value = 0.894). Considering the 64-MDCT, wide variations in the CNR values were seen when the kVp was elevated from 80 to 120 kVp, as well as one significant occurrence at 20 mAs and 0.625-mm thickness (*p* value < 0.639). In the 80-MDCT, there were insignificant increases in the CNR values at 10 and 20 mAs for each section during kVp increment from 80 to 120 kVp. Considering 50 mAs and 0.5- and 1-mm section thicknesses, there were inconsequential elevations occurring to the CNR values (*p* values = 0.51 and 0.77, respectively) for elevations from 80 to 120 kVp. In addition, further increments in the CNR values at 80 and 120 kVp were seen at 100 mAs and 0.5-mm section thickness (the *p* value = 0.81).

### 3.4. Impact of mAs on CNR Values

Increased mAs levels increased the CNR values, especially at higher section thicknesses, in all CT scanners. Several significant increases in CNR values occurred when the mAs was increased from 10 to 20 mAs, initially, and then to 50, 100 or 200 mAs (Table 7). Ultimately, there was inconsequential variations in the values of CNR between 10 and 20 mAs, especially at the lowest kVp and with progressively more thinner thicknesses (the *p* value > 0.1) in all corresponding CT scanners. Considering the 16-MDCT, there were inconsequential variations at 10 and 50 mAs as well as at 80 kVp, when using 0.625- and 1.25-mm thicknesses of the sections (*p* values = 0.914 and 0.244). Considering 120 kVp, there were inconsequential changes in the values of CNR at 10 and 20 mAs as well as with 0.625, 1.25 and 2.5 mm thicknesses (the *p* values = 0.76, 1 and 0.6, respectively). In the 64-MDCT, insignificant variations were present between images at 10 and 20 mAs and 120 kVp, with a 0.625-mm section thickness (*p* value = 0.781). In the 80-MDCT, there were insignificant variations between images at 10 and 50 mAs and 120 kVp, with a 1.25-mm thickness (*p* value = 0.643).

### 3.5. Impact of Section Thickness on CNR Values

Thicker sections produced increased CNR values in almost all the CT scanners. Several significant increases in CNR values occurred when the section thickness was increased from 0.625 to 1.25, 2.5 or 5 mm (Table 8). In the 16-MDCT, there was insignificant differentiation between the CNR values for the 0.625- and 1.25-mm section thicknesses at 20, 50 and 100 mAs and 80 kVp, as well as at 10, 20, 50 and 200 mAs and 120 kVp. There were also inconsequential differences when comparing the images with 0.625- and 1.25-mm section thicknesses at 20, 50, 100 and 200 mAs and 80 kVp, as well as at 10, 20, 50 and 200 mAs and 120 kVp. Inconsequential peaks were seen between the images utilising 0.625- and 2.5-mm section thicknesses at 10 and 50 mAs and 80 kVp, as well as at 10, 20 and 50 mAs and 120 kVp. In addition, there were inconsequential peaks between the images using 0.625- and 5-mm section thicknesses at 10 mAs and 80 and 120 kVp (Table 8). In the 64-MDCT, there were inconsequential variations in the CNR values in some places for the 0.625- and 1.25-mm thicknesses and for the 0.625- and 2.5-mm thicknesses at 10 and 20 mAs and 80 kVp. There were similarly inconsequential distinctions between images for the 0.625- and 1.25-mm thicknesses at 100 mAs and 80 kVp (*p* value = 0.138). There were also insignificant distinctions between the images of the 0.625- and 1.25-mm section thicknesses at 10 mAs and 120 kVp (Table 8).

Considering the 80-MDCT, inconsequential distinctions were present between the images of the 0.5- and 1-mm thicknesses at 20, 50 and 200 mAs and kVp of 80. In addition, there were inconsequential peaks between images of the 0.5- and 2-mm thicknesses when considered at 50 mAs and 80 kVp, as well as insignificant distinctions between images of the 0.5- and 1-mm section thicknesses at 20, 50, 100 and 200 mAs and 120 kVp. Inconsequential peaks were also seen between images of the 0.5- and 2-mm thicknesses at 120 kVp and 10 mAs (Table 8).

### 3.6. Correlation between Scanners Based on CNR Values 

**Figure 3:**
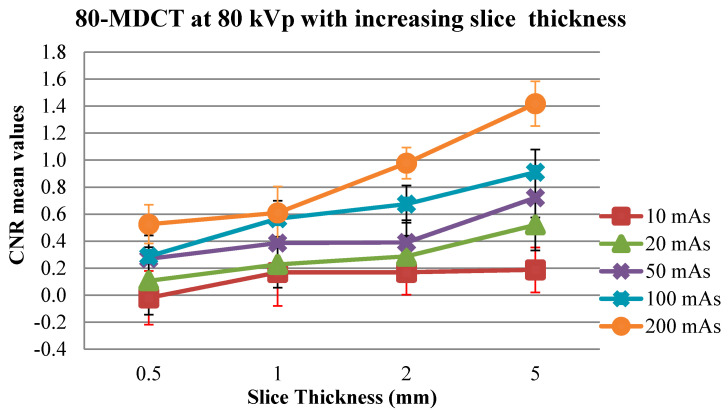
Thicker slice images resulted in higher CNR values at 80 kVp with different mAs levels for the 80-MDCT scanner. (Note the change in CNR values at 10 mAs among 1-, 2- and 5-mm slice thickness images and the change in CNR values at 50 mAs between 1- and 2-mm slice thickness images.)

With respect to the CNR values, the 64-MDCT worked as well as the other CT scanners, although the 16-MDCT produced higher CNR values than the 64-MDCT at 80 kVp and 10 mAs with a 1.25-mm section thickness. The 16-MDCT generally worked better than the 80-MDCT, although the 80-MDCT (Figure 2) produced better CNR values than the 16-MDCT at 80 kVp and 10 mAs with a 2.5-mm section thickness, at 80 kVp and 20 mAs with various cut thicknesses and at 80 kVp and 50 mAs with a 2.5-mm section thickness. Large variations were found between the 16-MDCT and 64-MDCT scanners, having certain exceptions: at 10 mAs and 80 kVp with 0.625- or 1.25-mm section thicknesses and at 50 mAs and 120 kVp with a 0.625-mm section thickness (Table 9). Basic differences were also seen in the CNR values between the 16-MDCT and the 80-MDCT scanners at low introduction factors: at 10 mAs and 80 kVp with 2.5- or 5-mm section thicknesses, at 20 mAs and 80 kVp with various section thickness; and at 50 mAs and 80 kVp with 0.625-mm/0.5-mm and 1.25-mm/1-mm section thicknesses. Change in CNR values at 10 mAs among 1-, 2- and 5-mm slice thickness images can be seen in Figure 3. The distinction in CNR values between the 64-MDCT and 80-MDCT scanners were continually basic. The 64-MDCT not only produced higher CNR values than the other scanners, it furthermore provided superior linearity in the CNR values, which increased with elevated mAs, kVp and section thickness.

## 4. Discussion

The effect of object size on CNR values was examined from various angles, in terms of scanner type, reconstruction calculation, contrast level of the object and mAs. The outcomes of the modifying factors—mAs, kVp and section thickness—on the values of CNR were also investigated, and the operation of the various scanners, based on the CNR values, were compared and correlated.

It was seen that the CNR values were considerably affected by adjusting the image reconstruction calculations. Although it was seen that the CNR values were high in the upper level in the soft-tissue reconstruction images in the 16-MDCT and 64-MDCT scanners, some CNR values were found to be higher in the standard reconstruction images than in the other types of algorithmic reconstruction images in the 80-MDCT scanner. This accords with the findings of several different studies [22,23]. As found by Kalender and Khadivi [1], changing the algorithmic reconstruction produces different trademark pixel sound estimates, which are characterised by the reconstruction. For example, a normal pixel sound for soft tissue is 62.1 HU, while, for standard and high-goal parts, they are 31.5 and 57.5 HU, respectively, for a 1- and 32-mm section thickness. The sound level of CT images is a significant factor, compared to the location of LCD objects [1,24]. The outcomes do not show any noteworthy changes in the CNR values among other object estimates, down to the smallest measurable object (5 mm). It was observed that the ability to identify objects depends on the differentiation standard of the object, as well as its size [19,20,21,22,23,24,25,26]. Step-by-step instructions to control undefined, minor lesions (e.g., nodules in CT lung screening) have become a significant concern. Although most very small nodules are benign, a few will end up being cancer-causing [27]. Some minor lesions, (e.g., small-cell carcinomas) grow in rapid increments, with a mean volume multiplying time of around 149 days [28]. Consequently, early intervention is vital to implement treatment [27]. The exact measurement and precise identification of the size of pulmonary nodules and various injuries are necessary in various clinical cases. This allows monitoring of the impacts of chemotherapy and injury development at follow-up, which may reveal risk [29]. An appraisal method based on estimated CNR values is not good enough to determine the consequences of object size on the capacity of the LCD.

Overall, a higher kVp produced superior CNR values in all the CT scanners. Specifically, there were critical elevations in CNR values when the voltage was elevated from 80 to 120 kVp. The impacts of kVp on the quality of image relative to the CNR values were as anticipated, with elevating kVp increasing photon entrance and the radiation portion when the other factors were notably fixed, even though the radiation portion is not straight with kVp. Subsequently, the noise is diminished and the CNR is increased [9,30].

Higher mAs levels mainly produced better CNR values, especially for thicker sections. As expected, the CNR is improved with increasing mAs because this decreases the image noise. The radiation portion is directly related to the mAs [31,32]. With thicker sections, the CNR values mostly got elevated. The impact of section thickness on the imaging ability of the CT scanners was anticipated because thicker sections decrease image noise so the image quality improves [33]. As indicated above, there were inconsequential differences in the CNR values for the 0.625-mm/0.5-mm and 1.25-mm/1-mm section thicknesses at all mAs levels, as well as between the 0.625-mm/0.5-mm and 2.5-mm/2-mm section thicknesses, especially at lower introduction factors. The noise elevates with thinner slices if the radiation dose is not allowed to escalate [9,30,33]. In any case, thinner sections give high-goal, isotropic image informational indices, and subsequently the through-plane, incomplete-volume averaging impacts are limited and the image is upgraded [1,34,35].

With respect to the CNR value estimations, the 64-MDCT scanner worked the best, whilst the 16-MDCT scanner worked better than the 80-MDCT scanner, for the most part. There were important and consequential differences between the 16-MDCT and 64-MDCT scanners. The specific properties and framework specifications of each scanner determined their imaging capabilities. The CT scanner manufacturer, model, geometry, tube details and locator configuration all determine the noise and image clarity, which all influence the imaging and image quality [9,26,36]. This study was restricted by using only one scanner arrangement, from one manufacturer, and by the smallest quantifiable object being 5 mm in diameter.

## 5. Conclusions

The LCD assessment technique, based on the CNR values, was sensitive to the quantification of the impacts of kVp, mAs and section thickness on image quality. This technique was also successful in assessing the impacts on image quality of various reconstruction calculations and objects at different levels, based on the CNR values. However, it should be noted that the smallest object size that could be estimated, using this technique with the Catphan^®^ 600, was 5 mm. Smaller objects could not be distinguished with any precision without estimating the outside of the object in order to determine the object’s mean CT value. In that respect, no notable CNR changes were found between various object sizes. Thus, LCD perceptibility is not only dictated by object differentiation, but also by object size. Therefore, this method is not an appropriate method to measure LCD detectability performance. It is also not possible for this phantom design to appraise an object of the same size and contrast at different position levels inside the phantom. It is a time consuming, inconvenient and vexatious as it necessitates scrutiny of an immensely vast quantity of data. Furthermore, authenticity of this technique is comparatively low as human eyewitnesses were not incorporated in the procedure. Thus, utilisation of Catphan^®^ 600 should not be considered as an appurtenant apparatus for image optimisation intentions and regularly based appraisals. In addition, volume CT dose index (CTDI_vol_), which is a systematised calculation of the radiation output coming out of a CT system, that permits users to find out the quantity of radiation emitted and equates the radiation output between various scanners, can be considered in future research. Another point to be mentioned is that the dose–length product (DLP), which is directly connected to the patient (stochastic) dangers, can also be utilised for future similar research.

## Figures and Tables

**Table 1:** Specifications of the CT Scanner.

	16-MDCT	64-MDCT	80-MDCT
Manufacturer’s name	GE Healthcare	GE Healthcare	Toshiba
Name of the product	LightSpeed 16	LightSpeed VCT 64	Aquilion Prime 80
Type of Detector	Solid-state polycrystalline ceramic scintillator	Solid-state ceramic	Solid-state Gd2O2S
Cell size of the Detector	0.625 mm	0.625 mm	0.5 mm
Coverage area	20 mm	40 mm	40 mm
Aperture of the Gantry	70 cm	70 cm	78 cm
Algorithm reconstruction	Filtered back projection, 2D back projection	GE property volume recon 2D back projection	Filtered back projection
Focal spot size	0.7 × 0.6	0.6 × 0.7	0.9 × 0.8
	0.9 × 0.7	0.9 × 0.9	1.6 × 1.4

**Table 2:** The Protocol parameters utilised for image acquisition.

Voltage kVp	80 and 120	
mAs Utilised	10, 20, 50, 100 and 200	
Section thickness	For the 80-MDCT (in mm)	0.5, 1, 2 and 5
	For 16-MDCT and the 64-MDCT (in mm)	0.625, 1.25, 2.5 and 5
Reconstruction algorithms used	Standard, lung and soft tissue	

**Table 3:** Mean CNR values of the images at 80 kVp. The mean values were obtained by averaging three identical exposures.

kVp	mAs	16-MDCT			64-MDCT			80-MDCT		
		Section Thickness	Mean	SD	Section Thickness	Mean	SD	Section Thickness	Mean	SD
80	10	0.625	0.24	0.18	0.625	0.26	0.29	0.5	−0.02	0.20
80	10	5	0.18	0.24	5	0.56	0.22	5	0.19	0.17
80	20	0.625	0.07	0.21	0.625	0.52	0.43	0.5	0.11	0.25
80	20	5	0.50	0.21	5	0.89	0.23	5	0.52	0.19
80	50	0.625	0.32	0.38	0.625	0.61	0.39	0.5	0.27	0.21
80	50	5	0.94	0.16	5	1.53	0.23	5	0.72	0.15
80	100	0.625	0.74	0.30	0.625	1.04	0.36	0.5	0.29	0.15
80	100	5	1.31	0.19	5	2.19	0.27	5	0.91	0.17
80	200	0.625	0.83	0.34	0.625	1.20	0.25	0.5	0.53	0.14
80	200	5	1.96	0.28	5	3.11	0.39	5	1.42	0.17

**Table 4:** Mean CNR values of the images at 120 kVp. The mean values were obtained by averaging three identical exposures.

kVp	mAs	16-MDCT			64-MDCT			80-MDCT		
		Section Thickness	Mean	SD	Section Thickness	Mean	SD	Section Thickness	Mean	SD
120	10	0.625	0.35	0.36	0.625	0.57	0.30	0.5	0.06	0.21
120	10	5	0.58	0.24	5	1.14	0.34	5	0.35	0.20
120	20	0.625	0.49	0.36	0.625	0.71	0.38	0.5	0.23	0.16
120	20	5	1.02	0.21	5	1.60	0.32	5	0.65	0.26
120	50	0.625		0.46	0.625	1.09	0.43	0.5	0.37	0.19
120	50	5	1.56	0.22	5	2.54	0.22	5	1.05	0.23
120	100	0.625	0.98	0.31	0.625	1.39	0.32	0.5	0.64	0.24
120	100	5	2.51	0.25	5	3.48	0.27	5	1.33	0.31
120	200	0.625	1.27	0.29	0.625	2.17	0.47	0.5	0.80	0.28
120	200	5	3.17	0.43	5	5.14	0.33	5	1.87	0.40

**Table 5:** Differences in CNR values (*p* values, student *t*-tests) between different object sizes at 1% contrast in each CT scanner.

Object Size	Object Sizes	Sig. (*p* Values, Student *t*-Tests)		
		16-MDCT	64-MDCT	80-MDCT
5	6	0.989	0.99	0.005
	7	0.228	0.999	0
	8	0	0.905	0.001
	9	0.145	0.799	0.002
	15	0.998	0.994	0
6	7	0.601	1	0.309
	8	0.004	0.998	0.999
	9	0.459	0.987	1
	15	1	1	0.333
7	8	0.308	0.982	0.537
	9	1	0.937	0.467
	15	0.472	1	1
8	9	0.436	1	1
	15	0.002	0.997	0.566
9	15	0.339	0.979	0.495

**Table 6:** The dissimilarities (student *t*-tests, *p* values) between images using the same quantity of mAs and section thicknesses with varying kVp in each CT scanner.

(J) Image Code (kVp-mAs-Section Thickness)	(I) Image Code (kVp-mAs-Section Thickness)	Sig. (*p* Values, Student *t*-Tests)		
		16-MDCT	64-MDCT	80-MDCT
80-10-0.625/0.5	120-10-0.625/0.5	1.000	0.050	0.923
80-10-5	120-10-5	0.031	0.000	0.254
80-20-0.625/0.5	120-20-0.625/0.5	0.011	0.639	0.603
80-20-5	120-20-5	0.000	0.000	0.508
80-50-0.625/0.5	120-50-0.625/0.5	0.000	0.000	0.769
80-50-5	120-50-5	0.000	0.000	0.000
80-100-0.625/0.5	120-100-0.625/0.5	0.894	0.024	0.000
80-100-5	120-100-5	0.000	0.000	0.000
80-200-0.625/0.5	120-200-0.625/0.5	0.005	0.000	0.018
80-200-1.25/1	120-200-1.25/1	0.002	0.000	0.000
80-200-2.5/2	120-200-2.5/2	0.000	0.000	0.000
80-200-5	120-200-5	0.000	0.000	0.000

**Table 7:** Dissimilarities (*p* values, student *t*-tests) between images utilising the same kVp and mAs with mAs variations in each CT scanner.

Image Code kVp-mAs-ST	Image Code kVp-mAs-ST	Sig. (*p* Values, Student *t*-Tests)		
		16-MDCT	64-MDCT	80-MDCT
80-10-0.625/0.5	80-20-0.625/0.5	0.395	0.203	0.310
	80-200-0.625/0.5	0.000	0.000	0.000
80-10-1.25/1	80-20-1.25/1	0.001	0.347	0.886
	80-200-1.25/1	0.000	0.000	0.000
80-10-2.5/2	80-20-2.5/2	0.188	0.099	0.120
	80-200-2.5/2	0.000	0.000	0.000
80-10-5	80-20-5	0.000	0.004	0.000
	80-200-5	0.000	0.000	0.000
120-10-0.625/0.5	120-20-0.625/0.5	0.760	0.781	0.166
	120-200-0.625/0.5	0.000	0.000	0.000
120-10-1.25/1	120-20-1.25/1	0.998	0.012	0.997
	120-200-1.25/1	0.000	0.000	0.000
120-10-2.5/2	120-20-2.5/2	0.060	0.002	0.109
	120-200-2.5/2	0.000	0.000	0.000
120-10-5	120-20-5	0.000	0.000	0.017
	120-200-5	0.000	0.000	0.000

**Table 8:** Dissimilarities (*p* values, student *t*-tests) between the images using the same kVp and mAs with varying section thickness in each CT scanner.

(I) Image Code	(J) Image Code	Sig. (*p* Values, Student *t*-Tests)		
		16-MDCT	64-MDCT	80-MDCT
80-10-0.625/0.5	80-10-1.25/1	0.004	0.348	0.029
	80-10-5	0.888	0.012	0.013
80-20-0.625/0.5	80-20-1.25/1	0.480	0.878	0.246
	80-20-5	0.000	0.007	0.000
80-50-0.625/0.5	80-50-1.25/1	1.000	0.029	0.228
	80-50-5	0.000	0.000	0.000
80-100-0.625/0.5	80-100-1.25/1	0.193	0.138	0.000
	80-100-5	0.000	0.000	0.000
80-200-0.625/0.5	80-200-1.25/1	0.065	0.020	0.384
	80-200-5	0.000	0.000	0.000
120-10-0.625/0.5	120-10-1.25/1	0.108	0.229	0.000
	120-10-5	0.075	0.000	0.001
120-20-0.625/0.5	120-20-1.25/1	0.773	0.003	0.434
	120-20-5	0.000	0.000	0.000
120-50-0.625/0.5	120-50-1.25/1	0.291	0.000	0.633
	120-50-5	0.000	0.000	0.000
120-100-0.625/0.5	120-100-1.25/1	0.003	0.000	0.980
	120-100-5	0.000	0.000	0.000
120-200-0.625/0.5	120-200-1.25/1	0.067	0.000	0.139
	120-200-5	0.000	0.000	0.000

**Table 9:** Dissimilarities (*p* values, student *t*-tests) between images of the same factors and section thicknesses utilising various CT scanners.

kVp	mAs	ST	Sig. (*p* Values, Student *t*-Tests)		
			16-MDCT × 64-MDCT	16-MDCT × 80-MDCT	64-MDCT × 80-MDCT
80	10	0.625/0.5	0.3961	<0.001	0.0009
80	10	5	0.0000	0.4788	0.0000
80	20	0.625/0.5	0.0003	0.3145	0.0009
80	20	5	0.0000	0.3921	0.0000
80	50	0.625/0.5	0.0153	0.3027	0.0015
80	50	5	0.0451	<0.001	0.0000
80	100	0.625/0.5	0.0050	0.0000	0.0000
80	100	5	0.0000	0.0000	0.0000
80	200	0.625/0.5	0.0004	0.0009	0.0000
80	200	5	0.0000	0.0000	0.0000
120	10	0.625/0.5	0.0276	0.0035	0.0000
120	10	5	0.0000	0.0018	0.0000
120	20	0.625/0.5	0.0395	0.0051	0.0000
120	20	5	0.0000	0.0000	0.0000
120	50	0.625/0.5	0.0635	0.0002	0.0000
120	50	5	0.0000	0.0000	0.0000
120	100	0.625/0.5	0.0002	0.0003	0.0000
120	100	5	0.0000	0.0000	0.0000
120	200	0.625/0.5	0.0000	0.0000	0.0000
120	200	5	0.0000	0.0000	0.0000
